# Silaffins in Silica Biomineralization and Biomimetic Silica Precipitation

**DOI:** 10.3390/md13085297

**Published:** 2015-08-19

**Authors:** Carolin C. Lechner, Christian F. W. Becker

**Affiliations:** 1 Swiss Federal Institute of Technology in Lausanne (EPFL), Fondation Sandoz Chair in Biophysical Chemistry of Macromolecules, 1015 Lausanne, Switzerland; E-Mail: carolin.lechner@epfl.ch; 2 Institute of Biological Chemistry, Department of Chemistry, University of Vienna, Währinger Straße 38, 1090 Vienna, Austria

**Keywords:** biomineralization, silaffins, diatoms, posttranslational modifications

## Abstract

Biomineralization processes leading to complex solid structures of inorganic material in biological systems are constantly gaining attention in biotechnology and biomedical research. An outstanding example for biomineral morphogenesis is the formation of highly elaborate, nano-patterned silica shells by diatoms. Among the organic macromolecules that have been closely linked to the tightly controlled precipitation of silica in diatoms, silaffins play an extraordinary role. These peptides typically occur as complex posttranslationally modified variants and are directly involved in the silica deposition process in diatoms. However, even *in vitro* silaffin-based peptides alone, with and without posttranslational modifications, can efficiently mediate biomimetic silica precipitation leading to silica material with different properties as well as with encapsulated cargo molecules of a large size range. In this review, the biomineralization process of silica in diatoms is summarized with a specific focus on silaffins and their *in vitro* silica precipitation properties. Applications in the area of bio- and nanotechnology as well as in diagnostics and therapy are discussed.

## 1. Silicon in Nature

Silicon is the second most abundant element in the Earth’s crust and associated with oxygen, silicates and silica (SiO_2_) constitute the most common compounds in the lithosphere [1]. The predominant types are the crystalline silicate minerals quartz and alkali feldspars, as well as amorphous biogenic silica [2]. In contrast to the numerous different inorganic silicon-containing compounds, there are no naturally occurring bioorganic substances clearly identified that require or contain silicon. Nevertheless, silicon is supposed to be an essential element for many biological systems [3,4].

In higher animals silicon is an essential nutrient required for proper growth and development [5,6]. In higher plants, silicon content ranges from 0.1% to 10% of the dry matter depending on the species [7]. Silicon is taken up by plants as silicic acid from soil and finally deposited as amorphous silica [8,9,10,11] However, silicon is not considered an essential element but as advantageous for plants since it is beneficial for plant growth, provides structural support for cell walls and mediates resistance of plants to biotic and abiotic stress [11,12,13].

Silicon is essential for a couple of specific biota including diatoms, siliceous sponges, radiolaria and silicoflagellates. These organisms require silicon for the production of siliceous structures, ranging from frustules, spicules and scales to various species-specific elaborate forms [14]. Exoskeletons made of silica are convenient for these organisms because they provide a large specific surface area, leading to high adsorption capacities and unique mechanical stability. Amongst the silica biomineralizing organisms diatoms are predominant and attract attention with their ornate silica frustules. Since silicon-deficiency not only effects diatom growth and cell wall formation but also interferes with metabolic processes [15], its essential role in diatoms is clearly confirmed. As a consequence, the molecular mechanisms and the biomolecules involved in silica formation in diatoms have attracted considerable attention. The currently available knowledge serves as a basis for biomimetic silica formation processes leading to functionalized silica for applications in (nano-) biotechnology with special emphasis on silica decorated with sensitive cargo molecules.

## 2. Silica Biomineralization in Diatoms

### 2.1. Diatom Biology and Cell Cycle

Diatoms are eukaryotic, unicellular organisms which are ubiquitously found in both marine and fresh water environments in all parts of the world as long as sufficient amounts of nutrients are present. Overall, there are more than 10,000 diatom species known to date, but it is estimated that more than 200,000 species exist worldwide [16,17].

**Figure 1:**
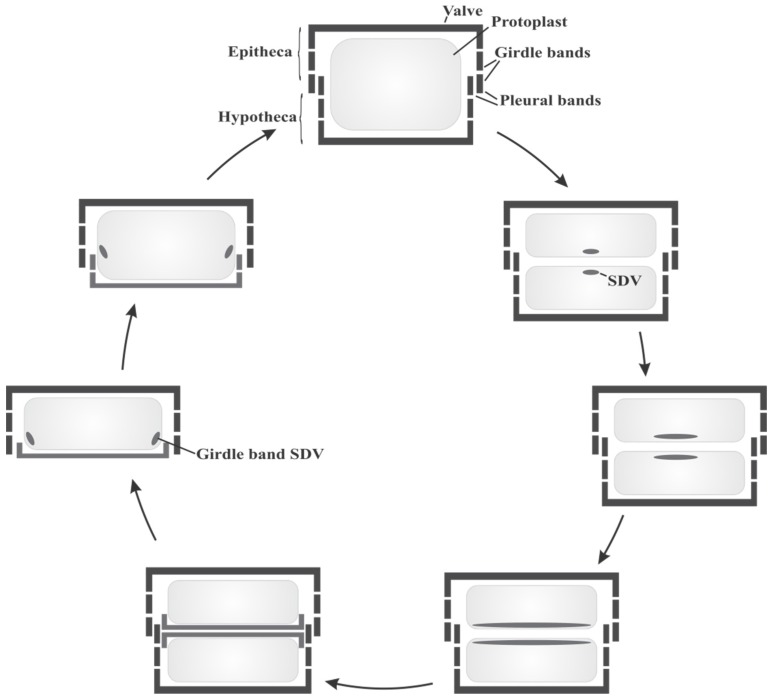
Schematic diatom cell cycle (modified from [19]).

Diatoms are usually microscopic organisms with cell sizes ranging typically from 10 to 200 µm [18]. The siliceous diatom cell walls are composed of two mirror-image halves, the epitheca and the hypotheca. Each theca consists of a capping valve and several girdle bands, which are silica strips running laterally along the axis of the cell. The last few girdle bands are summarized as the pleural band (Figure 1). The epitheca is slightly larger than the hypotheca, thus both fit into each other and together they completely enclose the protoplast. Whereas the valves generally display elaborate ornate silica architectures, the girdle bands are rather unstructured.

During their vegetative reproduction, which predominates over sexual reproduction [18], diatoms need to build up a new silica cell wall. The required silicon for the silica formation is taken up from their aqueous habitats predominantly as silicic acid [20]. The concentration of silicic acid ranges from 10–70 µM in surface waters and oceans [21], but intracellular concentrations can reach up to several hundred millimolar [22]. Diatoms actively enrich silicic acid by specific silicic acid transporter proteins (SITs) [23,24,25]. Sequence analysis of a variety of SIT genes have shown that the SIT proteins contain 10 transmembrane helices and a highly conserved sequence motif, GXQ (X = Gln, Gly, Arg or Met) [24], which is believed to be involved in binding and transport of silicic acid. The intracellular transport and storage of silicic acid in diatoms is still not well understood. Although silicic acid is soluble at neutral pH only until 2 mM, large pools of soluble silicon exceeding the level of silicic acid solubility have been observed in diatoms [22]. The molecular mechanisms or compounds involved to keep silicic acid soluble at high concentration and to prevent polycondensation of silicic acid are currently unknown.

The silica deposition finally takes place in a specialized compartment, the silica deposition vesicle (SDV). The occurrence of SDVs was shown in a variety of protists including diatoms, sponges and radiolaria, but the organelle could not yet be isolated for detailed biochemical analyses [26]. The membrane of the SDV, the silicalemma, possesses a membrane potential [27], and the pH of the SDV lumen is acidic [28]. Association of the SDV with the cytoskeleton via actin microfilaments and microtubules is important for silica molding, patterning and positioning of the SDV [29].

The cell cycle of diatoms starts with mitosis followed by cytokinesis, resulting in the division of the protoplast into two daughter cells (Figure 1). Before the daughter cells can separate a new silica frustule has to be generated. The silica formation for synthesis of new valves is initiated in the SDVs. With progressing silica precipitation the SDVs expand and once the silica synthesis is finished the newly formed valve is deposited on the cell surface of each protoplast by exocytosis of the SDV [26]. In addition, a stepwise synthesis of new siliceous girdle bands is necessary during cell growth to keep the protoplast enclosed in the silica shell. Depending on the species, girdle band formation can occur in different phases during cell cycle, either before or after cytokinesis [30,31]. Finally, the two sibling cells separate and during the interphase new silica girdle bands are required due to expansion of the protoplast. The girdle bands are each synthesized in separate SDVs and added to the frustule via exocytosis (Figure 1).

The diversity of silica nano-patterns of valves from different diatom species and the exact reproduction in each generation suggest a genomic encoding of molecular components that control silica formation and patterning. The fact of silica formation taking place in SDVs implies the occurrence of these components in the SDVs where they may act as initiators and nucleators of silica polycondensation and as structure directing molecules. To gain insight into the silica biomineralization process in diatoms, two major approaches are pursued: First, the analysis of diatom cell walls revealed organic molecules that are integral components of biosilica and potentially involved in the silica formation process. Second, sequencing and comparison of diatom genomes provided hints towards genetically encoded molecular processes of biosilicification. Several organic cell wall components, mainly proteins and polyamines, were identified that are associated with diatom biosilica and/or directly participate in silica formation.

### 2.2. Organic Constituents of Diatom Cell Walls and Their Role in Silica Formation

The siliceous frustules of diatoms are entirely surrounded by an organic matrix [32]. The initial identification of the unnatural amino acids 3,4-dihydroxyproline and ε-*N*,*N*,*N*-trimethyl-δ-hydroxylysine [33,34] and an overall analysis of diatom cell walls towards their amino acid content [35] clearly proved that proteins are inherent parts of diatom cell walls.

The first protein isolated from the cell wall of the diatom *Cylindrotheca fusiformis* was α1-frustulin, a glycoprotein of about 75 kDa. Frustulins were later shown to be general diatom cell wall proteins [36,37]. The glycoproteins of the frustulin family range from 30 to 200 kDa and share multiple acidic and cysteine rich domains of about 50 amino acids (ACR domains), which exhibit a specific affinity for Ca^2+^ ions. Frustulins are located in the organic matrix all over the cell wall, but they become associated with the silica only after silica formation and are therefore not considered to be involved in the biomineralization process [38,39]. Instead, a protective function for silica shells is suggested since silica dissolution of diatom frustules is accelerated by proteases [40] In addition, frustulins are able to chelate cadmium and might provide a barrier against potentially toxic metal ions [41].

Another group of proteins isolated from the silica cell wall of the diatom species *C. fusiformis* are the pleuralins. The former name “HEPs” (HF Extractable Proteins) indicates the strong binding of these proteins to the cell wall requiring dissolution of silica with anhydrous HF for release [38]. All pleuralins show a modular structure in which an *N*-terminal proline rich domain is followed by multiple repeats of the 90 amino acid PSCD domain, which is rich in proline (P), serine (S), cysteine (C) and aspartate (D), followed by variable *C*-terminal domains. The name “pleuralins” refers to the localization of these proteins to the pleural bands of the epitheca [31]. During cell division, pleuralins are deposited at the cleavage furrow and become associated with the newly formed pleural band of the hypotheca, thus possibly providing a protection to the protoblast.

In the diatom *Thalassiosira pseudonana* proteins with biochemical similarity to the pleuralins could be identified in the girdle band region [42]. These proteins showed no sequence homologies to the pleuralins, but they are highly acidic, contain several chitin binding domains and a putative RGD cell attachment motif. The proteins show apparent molecular masses of 130 (p130) and 150 kDa (p150) and their expression was highly upregulated in copper stressed cells [43]. The morphological effect of the Cu^2+^ stress was inhibition of the cell cycle but elongation of cell bodies as a result of additional synthesized girdle bands. Therefore, a function of the stress induced cell wall proteins p130 and p150 in the girdle band region is plausible, potentially during cell division as stabilizing and shielding proteins.

Remarkably, application of advanced atomic force and ion-abrasion scanning electron microscopic techniques revealed nano- and microscale structures and the occurrence of organic matrices in the girdle band region of *T. pseudonana* [44,45]. Chitin could be proven to be a major constituent of an organic scaffold that resembles the shape of the biosilica in the girdle band region [46]. These chitin based frameworks could serve as structural template for silica deposition [47] or as attachment site for other proteins. Notably, the stress induced cell wall proteins p130 and p150, which are also located in the girdle band region, contain multiple chitin binding domains [42]. Furthermore, another insoluble, but chitin-independent ring-shaped organic matrix, named microrings, could be identified in the girdle band region in *T. pseudonana* [48]. A class of proteins named cingulins is the integral component of these microrings. Cingulins are composed of highly repetitive structures with alternating KXXK-containing sequences as well as tryptophan and/or tyrosine rich regions. Most importantly, the microrings with embedded cingulins display activity in silica formation *in vitro* and the characteristic nanopatterns of the microrings are maintained after silicification. Since the nanopatterns of the microrings also resemble characteristic silica structures in the girdle band region of *T. pseudonana*, the assumption that preassembled protein-based organic templates act in general as scaffolds for the construction of the nanostructured silica cell walls of diatoms is reasonable. Besides the cingulins other biomolecules have been found to be associated with diatom cell walls and to be able to precipitate silica from a solution of silicic acid, specifically the silaffins and long chain polyamines (LCPAs).

**Figure 2:**
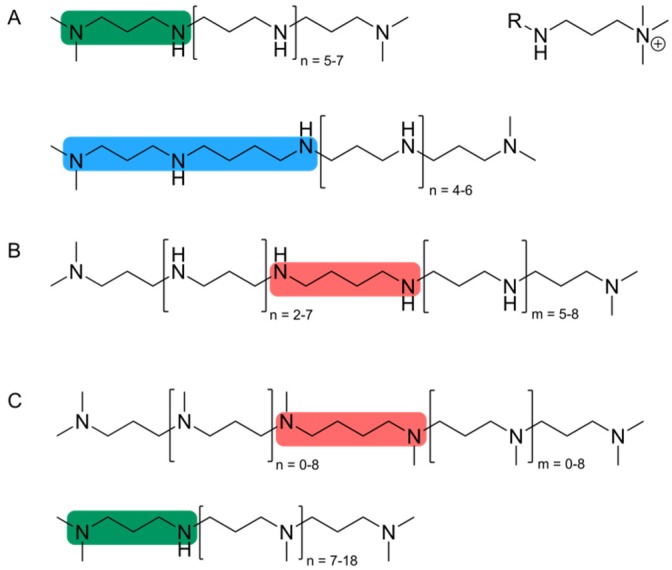
Structures of long chain polyamines (LCPAs) from different diatom species: (**A**) *T. pseudonana*; (**B**) *C. fusiformis*; (**C**) *S. turris*. The basis molecule is diaminopropane (green), spermidine (blue) or putrescine (red) (according to [52]).

LCPAs are major components of the silica cell walls of diatoms and released only after dissolution of silica [49]. All LCPAs have a common structure of linear oligo-propyleneimine chains attached to an amine-containing basis molecule but depending on the species from which the LCPAs originate, they differ in the basis molecule and in the number and degree of methylation of propyleneimine units (Figure 2).

The basis molecule is either putrescine, spermidine or 1,3-diaminopropane and the number of propyleneimine units ranges from 6 up to 20 [49,50,51,52]. The terminal nitrogen atoms of the propyleneimine units are often found to be dimethylated and a positive charge is sometimes introduced by quaternary amino groups [50,52]. The inhibition of polyamine biosynthesis in *T. pseudonana* resulted in incomplete silica valve formation and a reduced thickness of the silica [53]. In contrast, the addition of native LCPAs to preparations of cingulin-microrings increased the silicification rate [48]. Together with the finding that isolated LCPAs have the ability to trigger the rapid formation of spherical silica particles from a solution of silicic acid, a direct involvement of LCPAs in silica biogenesis in diatoms is obvious [49,54]. Silica precipitation activity of LCPAs *in vitro* strictly requires the presence of phosphate ions or other polyvalent anions such as pyrophosphate, sulfate or DNA in the reaction solution [54]. In addition, the species-specific LCPA structures hint towards an involvement of LCPAs not only in silica formation but also in patterning of specific silica structures in different diatom species [49,51,52].

Based on the cooperative action of polyamines and phosphate in silica formation, the idea of a biological phosphate-containing synergist being present during cell wall formation in diatoms was established. Recently, this hypothesis was confirmed by the discovery of the silacidins in the diatom *T. pseudonana* [55]. The precursor protein of the silacidins is highly repetitive and upon proteolytic processing releases peptides that are rich in aspartic and glutamic acid. The numerous serine residues within the silacidin sequence become phosphorylated converting them into strongly acidic and highly negatively charged peptides. A mixture of silacidins and LCPAs from *T. pseudonana* resulted in the precipitation of silica spheres from a solution of silicic acid with the size of silica spheres directly correlating with silacidin concentrations [55]. This effect has been described previously for phosphate ions [54] but much lower concentrations of silacidins are required to yield comparable amounts of precipitated silica. Phosphorylation of the serine residues has been proven to be essential for activity [56]. Additionally, the expression of silacidins is distinctly increased during silicic acid starvation [56]. Therefore, a function in rescuing silica formation in silicic acid depleted habitats is proposed for silacidins.

Besides LCPAs silaffins are the second major class of biomolecules identified from diatom cell walls. Silaffins are proteins combining both polycationic (polyamine) and polyanionic (phosphorylation) functionalities in one molecule that fulfill a substantial function in the molecular process of silica formation in diatoms.

## 3. Silaffin Proteins and Peptides

**Figure 3:**
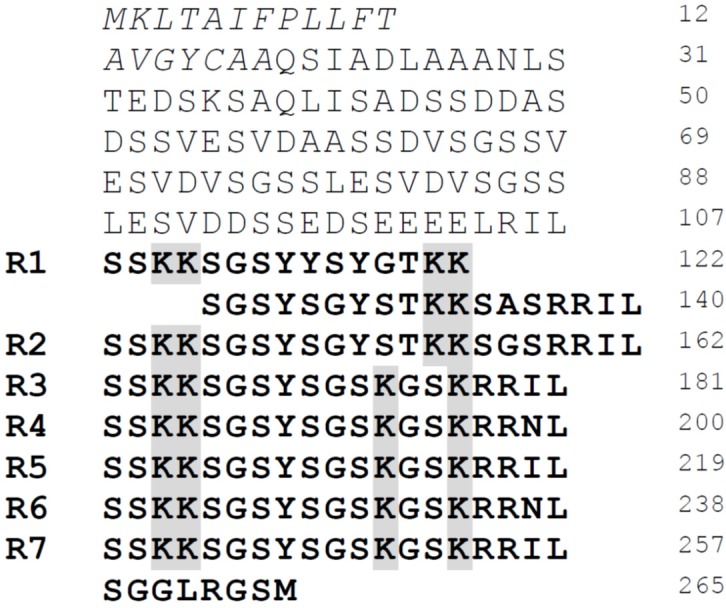
Primary structure of the silaffin precursor protein Sil1p. The signal peptide consisting of amino acids 1-19 is shown in italics and the repetitive units R1-R7 in bold. The lysine clusters in the repetitive C-terminal part are highlighted in grey. From Kröger, N.; Lorenz, S.; Brunner, E.; Sumper, M. Science 2002, 29, 584–586 [57]. Reprinted with permission from AAAS.

Silaffins were initially identified from the diatom *C. fusiformis* [57]. Extraction of the silica cell wall with anhydrous HF to release tightly bound organic material led to isolation of high molecular weight pleuralins [38] and proteins in the mass range of 4, 8 and 17 kDa. Based on their high affinity to silica, these proteins were named silaffins. The 4 kDa fraction was denoted silaffin-1A, the 8 kDa fraction was named silaffin-1B and the 17 kDa fraction silaffin-2. Based on preliminary sequence information of the silaffins, the corresponding gene *sil1* could be cloned from a *C. fusiformis* cDNA library. The open reading frame of *sil1* encodes the precursor protein Sil1p comprising 265 amino acids (Figure 3).

**Figure 4:**
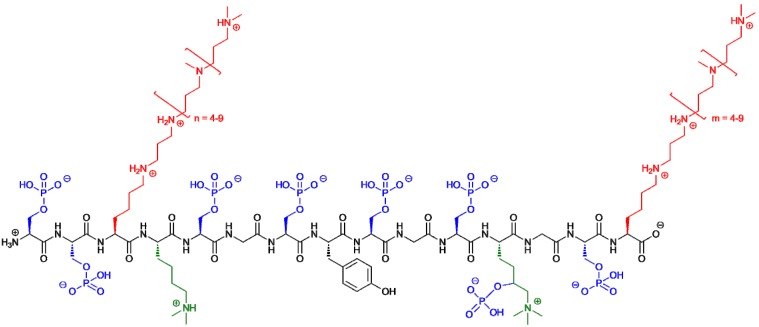
Chemical structure of native silaffin-1A_1_ from *C. fusiformis* with putative charges at pH 5 [59].

The protein contains an *N*-terminal signal sequence for translocation into the endoplasmic reticulum (ER) (amino acids 1–19) followed by an acidic *N*-terminal domain of yet unknown function (amino acids 20–107). The *C*-terminal part is strongly basic and highly repetitive (units R1–R7). Silaffin-1A and silaffin-1B both result from proteolytic processing of Sil1p. Silaffin-1B derives from peptide R1, whereas silaffin-1A can be further subdivided into silaffin-1A_1_, representing peptides R3–R7, and silaffin-1A_2_ originating from peptide R2 (Figure 3) [57,58]. The mature forms of silaffin peptides lack the *C*-terminal RRIL- and RRNL-sequences that are present in the repeat units R1–R7 (Figure 3 and Figure 4). Similar to the RXL motif *C*-terminal of the signal sequence in the precursor protein of Sil1p (Figure 3), also in other proteins associated with diatom silica, RXL motifs exist in precursor proteins located at the *C*-terminus of individual repeats, e.g., in frustulins, cingulins or silacidins [37,48,55]. Thus the RXL-sequence may serve as a general recognition motif for a specific endopeptidase in diatoms that processes precursor polypeptides by cleavage of the RXL motifs and releases the individual peptides.

Extensive analyses were necessary to reveal the complete chemical structure of silaffins due to numerous and extraordinary posttranslational modifications (PTMs) (Figure 4, Table 1) [57,58,59]. In silaffin-1A_1_ all lysine ε-amino groups are either di- or trimethylated or alkylated with *N*-methylated oligo-propyleneimine chains [57,58]. Polyamine-modification of lysine residues resembles LCPAs that are bound to putrescine and constitutes a unique PTM [49].

Changing the method for extraction of silaffins from diatom cell walls from HF to acidic aqueous ammonium fluoride preserved labile PTMs and gave evidence that all serine hydroxyl groups are phosphorylated. Also the trimethylated lysine residues become hydroxylated and phosphorylated at the δ-position [59]. Notably, the unique amino acid ε-*N*,*N*,*N*-trimethyl-δ-hydroxylysine has already been previously described as an organic component of diatom cell walls [34]. Together, all PTMs of silaffin-1A introduce a significant amount of both positive and negative charges rendering this peptide into a large zwitterionic molecule (Figure 4).

Silaffin-based peptides from *C. fusiformis* are capable of precipitating silica from a solution of silicic acid *in vitro*. The amount of precipitated silica is directly proportional to the amount of silaffin in the reaction and the silaffin peptides completely co-precipitate with the silica as long as silicic acid is present in excess [57]. The fully modified, native silaffin-1A_1_ efficiently precipitates silica from a solution of silicic acid at pH 5.5. In contrast, silaffin-1A carrying the lysine-modifications but lacking the serine phosphorylations is not able to initiate silica formation under these conditions. If phosphate anions are added to the reaction solution activity can be restored and maximal activity was observed at pH 5 [59]. The R5 peptide, a synthetic variant of silaffins with the sequence of the repetitive unit 5 of Sil1p but lacking any PTMs (Figure 3), has no silica precipitation activity below pH 6 [59,60]. Since the pH in the SDVs is acidic, modifications of lysine residues in silaffins seem to be essential for silica formation *in vivo* [28].

**Table 1:** Overview of silaffin variants identified from different diatom species.

Diatom Species	Silaffin	Posttranslational Modifications		Silica Precipitation Activity	References
		at Lysine	at Hydroxyl Amino Acids		
*C. fusiformis*	silaffin-1A and silaffin-1B	methylations and polyamine modification at ε-amino group; hydroxylation and phosphorylation at δ-position	phosphorylation	yes	[57,58,59]
				yes	
	silaffin-2		sulfation, glycosylation and phosphorylation	no	[61]
*T. pseudonana*	tpSil1p	methylations and polyamine modification at ε-amino group; hydroxylation and phosphorylation at δ-position	sulfation, glycosylation and phosphorylation	no	[62,63]
	tpSil2p				
	tpSil3p				
	tpSil4p				
*E. zodiacus*		methylations and polyamine modification at ε-amino group	not analyzed	not analyzed	[64]
*C. gracilis*		not analyzed	not analyzed	yes	[65]

Besides silaffin-1A and -1B, *C. fusiformis* diatoms express a third protein tightly associated with their cell walls, silaffin-2 (Table 1) [57]. Although the complete primary structure of silaffin-2 is not known the protein does not seem to be encoded by the *sil1* gene. The native form of silaffin-2 has a molecular weight of 40 kDa and is also highly posttranslationally modified [61]. Besides the lysine modifications known from the silaffin-1 variants, hydroxy-amino acids become phosphorylated, sulfated and glycosylated. The numerous sulfations and the abundant glucuronic acid in the carbohydrate modifications confer a strong anionic character to silaffin-2. Remarkably, and in contrast to silaffin-1A, native silaffin-2 has no activity in silica precipitation *in vitro*. This is most likely caused by the anionic modifications that overcompensate and inhibit the polyamine-modifications of lysine, which have been proven to be essential for silica precipitation activity [57,61]. However, a mixture of native forms of silaffin-2 and silaffin-1 or LCPAs is able to precipitate silica even under phosphate-free conditions *in vitro*. More interestingly different ratios of silaffin-2 and silaffin-1 result in silica precipitates with different morphologies and even porous silica block material could be obtained [61]. Therefore, the assumed function of silaffin-2 during cell wall biogenesis is rather regulation of silica formation and patterning than participating in the direct precipitation process.

Silaffin proteins could also be identified in other diatom species, e.g., *T. pseudonana* [62], *E. zodiacus* [64] or *C. gracilis* [65] (Table 1). In the silaffin proteins isolated from *E. zodiacus*, additional lysine derivatives with quarternary ammonium groups were identified, e.g., lysine derivatives alkylated with an aminopropyl moiety and further methylated a the ε-amino group of the lysine moiety [64]. Such modifications are most likely used to increase the affinity of silaffin peptides to the silica surface.

In *T. pseudonana* four silaffin precursor polypeptides are known, namely tpSil1p, tpSil2p, tpSil3p and tpSil4p (Table 1). Proteolytic processing of tpSil1p and tpSil2p results in low (20 kDa) and high (85 kDa) molecular mass isoforms [62,66]. None of these silaffin proteins shows sequence similarities to the silaffins from *C. fusiformis* but they are also rich in hydroxy-amino acids that become phosphorylated, sulfated or glycosylated. Based on these modification patterns silaffins from *T. pseudonana* are closely related to silaffin-2 from *C. fusiformis*. Both silaffin variants are unable to form silica *in vitro* by themselves but do so only in combination with LCPAs, they exhibit a regulatory function in silica formation that is comparable to silaffin-2.

A silaffin variant that is homologous to silaffin-1 from *C. fusiformis* with an ability to mediate silica formation *in vitro* has not yet been isolated from *T. pseudonana* even though similar lysine modifications have been found in tpSil3 and silaffin-1A_1_. The numerous (hydroxy-)lysine residues in tpSil3 either become dimethylated at the ε-amino group or alkylated with methylated aminopropyl units [63]. Modification of lysine with longer polyamines was not observed. Most of the lysine residues in tpSil3 are arranged in clustered tetrapeptides with KXXK motifs [63]. The clustering and arrangement of lysine in KXXK motifs is also observed in silaffins and cingulins from *C. fusiformis* [48,57,58,63] and might serve as a general recognition sequence for specific enzymes that introduce the extraordinary PTMs [63]. More importantly, clustering of several lysine residues in combination with phosphoserines in the tpSil3 protein was found to be crucial for targeting of silaffins to biosilica [67]. The *N*-terminal signal peptide of silaffins mediates co-translational import into the ER, together with the Golgi, the location for phosphorylation and glycosylation. Indeed, a specific silaffin kinase associated with the ER and Golgi membranes was identified in *T. pseudonana* [68]. Expression of the tpSTK1 kinase, a 60 kDa protein including an *N*-terminal signal peptide for import into the ER, is significantly upregulated during silica valve formation. tpSTK1 shows specific activity in phosphorylation of silaffin substrates, but not of silacidins, indicating a preference for substrates with basic isoelectric points. Accordingly, the intracellular transport pathway of silaffins starts with co-translational import of the silaffin precursor proteins into the ER, where they become phosphorylated [67]. The location of further modification and processing reactions as well as details on the transport from the ER to the SDV where silica formation takes place remain unclear.

## 4. Silaffin- and LCPA-Mediated Silica Formation in Diatoms

### 4.1. Chemical and Mechanistic Aspects of Silica Formation

**Figure 5:**
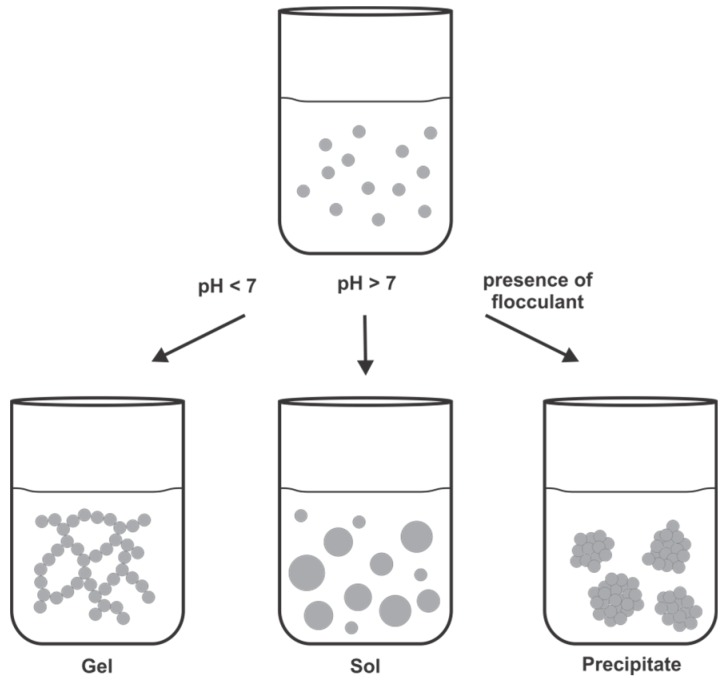
Formation of a silica gel, sol or precipitate from a colloidal silica sol depending on the reaction conditions.

Amorphous silica is formed by a complex inorganic polymerization process with orthosilicic acid as the monomeric building block. Solubility of monosilicic acid Si(OH)_4_ is limited to a concentration of 2 mM in aqueous solutions of neutral pH and deprotonation at pH values above 9 gives silicate anions SiO(OH)_3_^−^ [69]. Nucleophilic substitutions between silicate anions and silicic acid molecules lead to condensation reactions that form siloxane bonds (Si–O–Si). Silicic acid molecules react to form dimeric, trimeric and tetrameric species that further condense with monomers to form highly dense, branched polysilicic acid species. These colloidal silica particles have sizes in the nanometer range. Depending on the pH and the presence of salts or other additives, such a silica sol can undergo a variety of reactions (Figure 5). At pH values below 7 there is only weak electrostatic repulsion between the colloidal silica particles due their uncharged surfaces and the colloidal particles aggregate to fibrillar, branched chains forming a gel. At pH values above 7 negative charges on the surfaces of the colloidal silica particles dominate and induce electrostatic repulsion. Therefore colloidal silica particles form a stable sol and the particles grow by the Ostwald ripening process [69,70]. The addition of a cationic flocculant to a silica sol leads to fast precipitation of silica particles. Cationic species adsorb to silica surfaces and bring them close together resulting in coagulation of particles. Alternatively, cationic species, e.g., polyamines are suggested to stabilize the pentavalent transition state of the condensation reaction between silicic acid molecules and therefore further promote silica flocculation [71,72,73].

Formation of silica in diatoms occurs under acidic conditions in the SDV and the occurrence of colloidal silica could be proven in nascent diatom cell walls [30,74,75]. Since silica biomineralization in diatoms is much faster than abiotic silica formation, a biological flocculant is believed to assist the silica polycondensation. LCPAs and silaffins have been shown to be directly involved in the molecular processes that lead to biogenesis of the elaborate patterned silica frustules. Both, LCPAs and silaffins are highly cationic compounds, and can serve as flocculant for negatively charged silica nanoparticles.

### 4.2. LCPA-Induced Silica Formation Based on Phase Separation

Based on the physicochemical properties of amphiphilic LCPAs a phase separation model for silica formation has also been proposed [76]. LCPAs are able to rapidly precipitate silica from a solution of silicic acid *in vitro* but polyvalent anions, such as phosphate, are required for this reaction [54]. The LCPAs in a phosphate buffered solution can undergo a phase-separation process and form polyamine-polyanion-rich microdroplets. Silicic acid in the aqueous interface between the droplets is then rapidly polymerized to silica mediated by the polyamines. Species-specific patterns observed in diatom biosilica most likely result from variations in the polyamine droplet size [76]. The microscopic phase separation of synthetic polyamines in aqueous solutions is induced by the addition of multivalent anions and essential for polyamine-mediated silica precipitation [77]. Silica precipitation with polyamines in the presence of increasing phosphate concentrations produced spherical silica particles with increasing diameters and the process strongly depends on the pH [54,77,78]. With synthetic LCPAs, structurally based on natively occurring LCPAs, a clear connection between morphologies of silica precipitates and the structure of polyamines with respect to polyamine chain length and degree of *N*-methylation was demonstrated [79]. Altogether, the structure of the polyamines and the ratio of polyamine to phosphate seem to define the size of microdroplets and thus the final size of silica spheres. In diatoms highly acidic phosphorylated silacidins could be identified as the potential native source of polyanions that assist phase separation of LCPAs [55,56]. Since silacidin concentration directly influences the size of the resulting silica particles these peptides are not only serving as cross-linking polyanions that guide assembly of polyamines and assists in silica formation but they are also involved in the control of silica morphology in diatoms.

### 4.3. Silaffin-Induced Silica Precipitation

**Figure 6:**
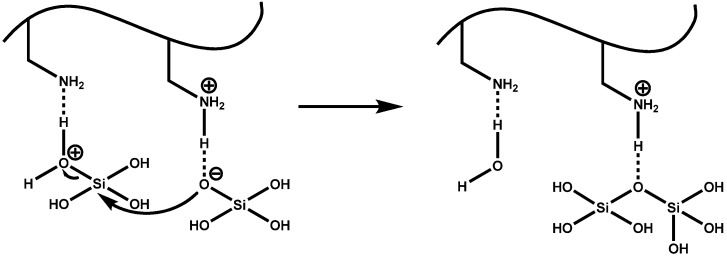
Proposed mechanism for poly-condensation reaction of silicic acid facilitated by the polyamine groups present in silaffin peptides.

In case of the silaffins a model for their silica formation activity that is in agreement with the model for silica formation by LCPAs was proposed [76]. Due to the polyamine modifications and the numerous phosphorylations, native silaffin peptides are zwitterionic and self-assemble in solution via electrostatic interactions with the numerous phosphate groups serving as an intrinsic anion source [59]. Polycationic silaffins lacking the native phosphorylations require the addition of divalent anions that assist as ionic cross-linkers in the self-assembly process [59]. Peptide self-assembly induces a microscopic phase separation and a high local concentration of amino groups in the aqueous solution. Since amines and polyamines have been generally shown to promote the condensation of silicic acid and thus the formation of silica [71] the amino groups in silaffins are supposed to act as acid-base catalysts that facilitate formation of siloxane bonds in this model [80]. Deprotonated amino groups accept a proton from a silicic acid molecule resulting in the formation of a reactive silanolate group. During the nucleophilic attack of this group at a second silicic acid molecule, protonated amino groups facilitate the release of water from the attacked molecule by protonation (Figure 6). Advancing condensation between silicic acid monomers and the colloidal silica particles in the sol finally results in precipitation of silica.

In native silaffin peptides, the unique polyamine modifications (Figure 4) have proven essential for their silica precipitation activity and are supposed to mediate siloxane bond formation [57]. Nevertheless, a synthetic silaffin peptide, the repetitive unit R5 of the silaffin polypeptide from *C. fusiformis* lacking any PTMs (Figure 3), has also been shown to efficiently trigger silica precipitation *in vitro* [57,59,60,81]. Here the interplay between the positively charged ε-amino groups of the lysine residues in the peptides and negatively charged phosphate ions (as required buffer components) are supposed to mediate formation of siloxane bonds resulting in precipitated silica [80]. The conclusive proof of the essential role of the lysine residues in the R5 peptide was provided with a R5 variant, in which all lysine residues were replaced by alanine resulting in complete loss of silica precipitating activity [82].

In addition, an appropriate arrangement and spacing of the amino groups in their peptidic context should stabilize the transition state in silicic acid poly-condensation and thereby enhance silica formation. The arrangement of lysine residues may also act as a template for silica patterning and lead to changes in silica morphology. Scrambled R5 variants (with random order of the native R5 amino acids) are still fully active in silica precipitation due to the presence of the ε-amino groups [82]. However, the disturbed morphology of the resulting silica spheres highlights the importance of a well-balanced pattern of functional groups within the native silaffin sequence.

The native PTMs of silaffins are not only essential for their silica precipitating activity but they are also involved in the nano-patterning of the biosilica leading to the ornate silica shells of diatoms. To systematically study the structure-function relationship of individual PTMs of silaffins, we recently presented the synthesis of silaffin variants carrying different side chain modifications based on naturally occurring PTMs, such as trimethylation, polyamine attachment and phosphorylation [83]. A distinct influence of the peptide modifications on both silica precipitating activity and morphology of the resulting silica material was observed. These results emphasize the intricate interplay of attractive and repulsive electrostatic interactions between peptide side chains and silicic acid molecules during silica formation [83].

In general, the arrangement of different modifications in the context of the native silaffin sequence leads to highly complex peptides that can self-assemble, mediate silicic acid poly-condensation and play a major role in nano-patterning of diatom cell wall silica. Combining our current knowledge about the sequence and modification dependence of silaffin peptide precipitation properties, the design of application-oriented biomimetic silica precipitation agents for different purposes is within reach and will be discussed in more detail below.

## 5. Biotechnological Applications of Silica

The scope of applications of silica is tremendous due to its unique chemical and mechanical properties and its easy availability. Besides classic applications of silica as absorbent, stationary phase in liquid chromatography, catalysis or general filling material, it also finds increasing implementation as an additive in cosmetics and in the food industry since it is generally recognized as safe [84,85]. The progress in syntheses of silica materials with defined structures and properties and the development of novel routes for biomimetic silica formation have prompted the application of silica based materials particularly in biotechnology and biomedicine [86,87,88,89].

### 5.1. Synthetic Silica Materials

Porous silica is a suitable material for biotechnological and medical applications because of its chemical inertness and biocompatibility. Nevertheless, the small diameter of the micropores (0.5–1 nm) in naturally occurring porous silica materials (e.g., zeolites) exclude these materials from biotechnological applications that typically involve proteins or other biomolecules larger than these pores. A breakthrough with respect to biotechnological applications was achieved with a novel type of highly ordered mesoporous silica-based materials, named MCM-41 (Mobile Crystalline Material-41) [90,91]. The synthesis of such highly ordered mesoporous silica materials is based on the cooperative micellar self-assembly of cationic surfactants or block-copolymers and anionic silicate precursors into ordered silicate-surfactant composites. The surfactants serve as templates for the poly-condensation of silicic acid and calcination removes the surfactant template, resulting in pure, mesoporous silica material. The characteristics of the porous silica material, *i.e.*, morphology, pore volume and diameter, are determined by varying and use of different combinations of silica sources or surfactants, pH and temperature [92]. MCM-41 based silica materials and others are characterized by uniformly sized mesopores formed by amorphous silica walls and consequently a high surface area, large pore volume and tunable pore sizes.

Besides the use of of mesoporous silica as stationary phases in HPLC, as filling material or in catalysis [93], an initial study has proven that MCM-41 mesoporous silica nanoparticles (MSNs) can be loaded with the anti-inflammatory drug ibuprofen and enable a sustained release [94]. This finding led to an enormous increase in the application of MSNs as advanced drug delivery systems, further promoted by the fact that MSNs are readily internalized by eukaryotic cells. Even the uptake efficiency can be tuned by morphology of the silica materials and by surface functionalization of the nanoparticles [95,96,97].

Mesoporous silica nanoparticles can generally be loaded with cargo molecules either by covalent attachment to a functionalized silica surface [98,99], or more commonly, by the immersion loading method. Here the MSNs are soaked in a solution of the cargo molecule [100], which can lead to very variable loading efficiency, depending on various factors including the functionalization of the silica surface [101]. The accessibility of MSNs with large pore sizes allows loading of bulky biomolecules such as peptides, proteins or even antibodies into the silica matrix [102,103,104]. The interaction of such cargo molecules with MSNs and therefore a fine-tuning in loading and release characteristics can be adjusted by selective functionalization of the inner core silica or the outer particle surface [105,106]. Functionalization of a silica surface can be achieved by co-condensation of functional molecules during synthesis or by post-synthetic grafting of functionalized silanes and can result in an effective control of drug release [106,107,108,109]. The introduction of organic functionalities to the silica surface of MSNs facilitates additionally the attachment of targeting moieties and extends the applications of MSNs to targeted therapies. Surface modification of MSNs, e.g., with folic acid [110,111], mannose [112], lactobionic acid [113], cyclic RGD peptide [114], transferrin [115] or antibodies [116], provided specific targeting to cancer cells. Specific targeting of drugs to their target location and a controlled release of the drug will certainly entail a reduction of the applied drug doses and reduce unwanted side effects of drugs.

**Figure 7:**
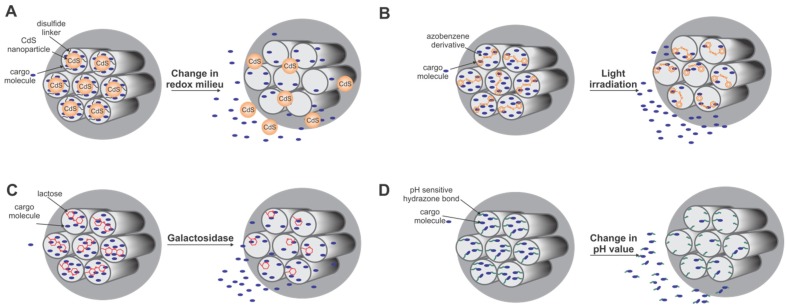
Stimulus-responsive systems for controlled release of cargo molecules from mesoporous silica nanoparticles. (**A**) redox-responsive release (adapted from [119]); (**B**) light irradiation (adapted from [125]); (**C**) enzymatic removal of gatekeeper (adapted from [121]); (**D**) pH-sensitive release (adapted from [129]).

Avoiding pre-mature release of loaded cargo from MSNs is possible by sealing the pores, e.g., by deposition of a lipid bilayer on functionalized MSNs to form a core-shell hybrid system [117,118]. To liberate cargos from MSNs only in response to a specific trigger effect, sophisticated stimulus-responsive systems have been developed. All of these systems have in common the containment of cargo molecules in the silica material by sealing the opening of the pores with a cap or “gatekeeper” (Figure 7). One prominent approach is the use of redox responsive gatekeepers, since release of cargos after endocytosis of the nanoparticles in the intracellular, reductive environment is achieved. Different gatekeepers, such as cadmium sulfide (CdS) nanoparticles [119] (Figure 7A), collagen [113] or a cross-linked polymeric network [120] were linked to a functionalized silica surface. Intracellular thiols readily cleave the disulfide bonds and detach the gatekeepers from the entrance of the pores, resulting in the release of the encapsulated cargo molecules.

Another strategy for stimulus responsive release is based on enzymatic removal of a gatekeeping agent, e.g., cleavage of lactose caps by β-galactosidase [121] (Figure 7C), proteolysis of a peptide shell [122], tryptic digest of avidin from a biotin-avidin cap system [123] or removal of a duplex DNA cap by endonucleases [124]. The latter two are also examples for dual stimuli-responsive systems since they allow cap removal not only enzymatically, but also via temperature shifts. Application of light-sensitive molecules as gatekeepers empowers spatiotemporal control over drug release. Examples include azobenzene derivatives as gatekeepers [125,126] (Figure 7B), photosensitizers that mediate opening of a nanoparticle supported membrane [127], or a red-light based photoactivation approach [118]. Different approaches use competitive displacement [128] or changes in pH [129,130] (Figure 7D) as trigger for stimulus responsive release of cargo molecules from mesoporous silica materials.

Altogether, multifunctional MSNs combining efficient cargo loading, a strategy for containment and stimulus-responsive release of cargo, and a moiety for targeting to a desired location are major constituents for establishing an advanced drug delivery system.

Besides the wide usage of MSNs in drug delivery, MSNs are also excellent matrices for biosensing applications. The high porosity, the large surface area and pore sizes of mesoporous silica allows detection of even very large bio-analytes and the incorporation of a high amount of sensor molecules into the porous matrix. These advantages lead to an improved detection limit and a faster diffusion of the analytes through the mesopores to the sensor molecule providing a shorter response time. Effective sensors for glucose, H_2_O_2_, NO_2_, ATP or neurotransmitters were generated by immobilization of sensor molecules on mesoporous silica materials [131,132,133,134,135,136]. The various possibilities for functionalization of mesoporous silica materials also enable development of further diagnostic or imaging applications [114,137].

However, despite the tremendous progress in generating tailored mesoporous silicas and the many examples for their application, the major disadvantages are complicated syntheses and harsh reaction conditions. Furthermore, the elaborate, hierarchically structured silica architectures observed in nature are currently still out of reach for chemical silica syntheses. The ability to form even complex nanostructured silica under ambient, very benign conditions draws the attention to biogenic or biomimetically formed silica.

### 5.2. Diatomaceous Earth and Biogenic Diatom Silica

Geological deposits of fossilized skeletons of diatoms are referred to as diatomaceous earth, diatomite or kieselguhr. The main component is silicon dioxide besides minor quantities of aluminum and iron oxide, whereas the exact composition depends on the place of origin [138]. Because of the high content of diatom silica frustules, diatomaceous earth has specific properties such as low density and conductivity but a large surface area and adsorption capacity due to porosity. Owing to these characteristics, diatomaceous earth has for a long time been extensively used as adsorbent [139], natural insecticide [140], insulating material [141], filter aid in wastewater treatment [142,143], or as catalyst carrier for photocatalytic reactions [144,145].

Due to the highly porous, hierarchically nanopatterned architecture, diatom silica also has a remarkable mechanical stability and displays photoluminescence and properties of a photonic crystal [146,147,148]. This unique combination of properties, produced under physiological conditions, has led to even more advanced applications as discussed above for synthetic silica materials.

Frustules of diatoms can be used as templates for the production of metal surfaces with elaborate patterned features that are valuable for Surface Enhanced Raman Spectroscopy (SERS). Coating of purified diatom frustules with metal layers followed by dissolution of silica leaves metallic materials that reflect the exact nanopattern of the silica template [149].

Furthermore, silica frustules of diatoms are useful templates for conversion of silica in other materials, such as nanocrystalline silicon or amorphous graphite [150,151]. Reaction of the diatom silica with gaseous magnesium and subsequent removal of the MgO with diluted hydrochloric acid at 650 °C gives nanocrystalline silicon. Otherwise, from the SiC that results from reaction of diatom biosilica with methane, the silicon can be removed with Cl_2_ at 950 °C, resulting in pure carbon. In both examples, the nanoscale structures of the diatom silica are preserved [150,151]. The transformation of diatom silica templates significantly increases the specific surface area of the formed highly porous silicon or carbon materials, thus providing materials with possible applications in sensing, catalysis, (bio-) chemical separation or energy storage and harvesting. Alternatively, the mineral composition of diatom frustules can be changed. GeO_2_ or TiO_2_ can be incorporated into the nanostructure of the silica cell wall by addition of Ge(OH)_4_ or TiCl_4_ to the culture medium and exploitation of diatoms as *in vivo* catalysts. Such Si-Ge composite materials could be applicable in the fabrication of electroluminescent display devices, battery electrodes or dye-sensitized solar cells [152].

Diatom shells display an efficient visible photoluminescence emission strongly dependent on the environmental conditions. This luminescence can be quenched or enhanced by several gaseous substances, thus diatom biosilica can be used as material in optical gas sensing applications [153,154]. In addition, functionalization of intact diatom frustules with an antibody was shown to enable biosensing of complimentary antigens via photoluminescence [155].

Tethering of biomolecules to biosilica is achieved by silanization of the surface silanol groups and coupling of a heterobifunctional crosslinker followed by the attachment of the biomolecule, e.g., an antibody [156]. Other potential applications of biosilica as carrier for covalent bound antibodies include immunoprecipiation and immunoisolation [157], or the development of a diagnostic device for electrochemical detection of biomolecules [158].

The possibility to selectively modify purified diatom biosilica with biological molecules also enables the development of silica microcapsules for targeted drug delivery. The silica shells of diatoms are highly convenient as an inert biomaterial carrier for drug delivery applications. Their hollow body structures and the micro- and nanoscale porosity allow straightforward loading and sustained release of hydrophobic and hydrophilic cargo molecules [159,160]. Functionalization of diatom silica surfaces with different organosilanes allows tuning of the drug loading and release properties [161,162].

In a different approach, living diatoms are exploited to achieve immobilization of an active enzyme in the biologically produced nanoporous silica material [163]. Silaffin proteins are involved in the silica formation process in diatoms and become tightly associated with the newly deposited frustules. Genetic fusion of a target enzyme with a silaffin gene and expression of such a fusion protein results in immobilization of the enzyme in the silica matrix. The enzyme containing biosilica can be gently purified and since the enzymes are not completely enclosed within the silica, their activity is largely retained whereas protein stability is significantly increased. This method has also proven applicable to oligomeric enzymes or enzymes that require posttranslational modifications or cofactors for activity [164]. Apparent advantages of this method include that the physiological conditions are beneficial for protein integrity and that the protein encapsulation in the nanostructured biosilica provides an ideally suited, mechanical stable and resistant matrix that ensures simultaneously substrate accessibility.

### 5.3. Biomimetic Silica

Besides synthetic silica and biogenic silica, biomimetic silica formation has gained more attention due to the possibility to combine mild reaction conditions with control over silica structure and relatively simple cargo loading. Approaches toward bioinspired and biomimetic silica formation were stimulated by the progress in unraveling the molecules involved in silica biomineralization processes in nature. A number of biomolecules could be identified including silaffins and LCPAs from diatoms that proved to be directly involved in the molecular processes leading to silica formation [49,57]. Investigations of their structures and their functional role in silica precipitation revealed chemical and physical prerequisites of biomolecules for activity in silica precipitation. An overall cationic character, more precisely lysine residues in case of peptides and proteins, and the ability to self-assemble in solution have been validated as required features of biomolecules that can induce silica formation. Transferring these insights of biological silica formation to *in vitro* processes enabled the development of novel silica materials with defined structures and properties under mild, physiological reaction conditions.

#### 5.3.1. Formation of Biomimetic Silica with Different Silica-Precipitating Agents

(Bio-)Molecules that have been successfully used in biomimetic silica formation include peptides and proteins such as poly-l-lysine (PLL), poly-l-arginine (PLA), the R5 peptide, lanreotide, block-copolypeptides and lysozyme, diverse polyamines such as polyallylamine (PAA), polyethyleneimine (PEI) or amine-terminated dendritic structures (Table 2).

The use of silica-precipitating molecules at room temperature results in formation of amorphous silica that can adopt a large variety of morphologies, depending on the exact conditions and additives (Table 2). Spherical silica is the thermodynamically preferred structure and is readily obtained using native silaffins [57,59], the R5 peptide [60,81,82] and linear or cyclic amines [165,166,167,168]. Nevertheless, the morphology of precipitated silica can be influenced by variation of the reaction conditions or by the chemical and structural nature of the mediating additive. Using the R5 peptide as a silica precipitating agent, different morphologies deviating from the common silica spheres that are obtained under static reaction conditions, e.g., fibrillar or arch-shaped structures can be achieved by keeping the reaction mixture in motion [169]. An externally applied electrostatic or hydrodynamic force field was shown to induce fiber-like structures [170]. The presence of polyhydroxyl compounds, e.g., glycerol or sucrose, prompted the formation of nanostructured sheet-like silica precipitates [170]. Purified predominantly cationic silaffin-1A results in the formation of spherical silica particles with diameters from 500 to 700 nm but in mixture with mainly anionic, glycosylated silaffin-2 the silica material changes to a composite of small silica nuclei [57]. Polycationic peptides such as poly-l-lysine (PLL) and poly-l-arginine (PLA) are well-known to precipitate silica from a solution of silicic acid [72,171]. Under static conditions PLL has been shown to trigger the formation of silica spheres and hexagons, whereas perturbation of the reaction mixture or application of an electrostatic field change silica morphologies to fiber-like, dendrite-like or ladder shapes with periodic voids [170,171,172]. The hexagonal silica plates observed by PLL-mediated silica formation are closely linked to PLL chain length and self-assembly of PLL into a helical conformation in the presence of phosphate anions [173,174]. A different study showed that PLL assembles into microspheres in the presence of citrate as counterion and the surface of these microspheres can be coated with silica [175]. Using large molecular weight PLL, it was recently shown that mesoporous silica materials with pore size distributions comparable to synthetic MCM-41 can be obtained without harsh reaction conditions [176]. Notably, application of bio-inspired, arginine-based surfactants in silica formation followed by calcination gives porous silica materials [177].

Since self-assembly emerged to be a prerequisite for silica formation activity, different molecules were considered as structure directing silica formation agents. Block copolypeptides such as poly(l-cysteine_30_-b-l-lysine_200_) self-assembled into structured aggregates in solution mediate the formation of ordered silica morphologies. The oxidation state of the cysteines in these polypeptides affects the self-assembly and the morphology of the resulting silica material can encompass hard silica spheres (under reducing conditions) as well as silica in packed columns (with oxidized copolypeptide) [178]. The synthetic octapeptide lanreotide is known to self-assemble into nanotubes [179] and when used as template double-walled silica nanotubes can be produced [180]. Also amphiphilic peptides such as A_6_K or V_6_K, which self-assemble into nanotubes or lamellar stacks can be used as organic templates in biomimetic silica formation. The presence of anions is necessary in these systems and depending on the peptide and anion composition or on external forces different silica morphologies could be obtained (Table 2) [181]. Recently, hybrid silica nanoparticles were generated with elastin-like polypeptide (ELP) micelles. Amphiphilic ELPs were genetically fused with the R5 peptide sequence at the hydrophilic terminus to form ELP-R5 diblock copolymers. These polypeptides self-assemble into micelles and serve as effective templates for biomimetic silica formation mediated by the R5-peptide. This method allows for facile loading of target molecules to silica particles for the development of drug delivery systems [182].

In addition to the peptide-based silica precipitating agent discussed above, amines and polyamines are generally able to precipitate silica due to their polycationic character [71]. The morphology of the silica material obtained e.g., from polyallylamine hydrochloride (PAA) strongly depends on the reaction conditions and formation of fiber-like structures is possible under externally applied shear stress [183,184]. The size of the silica particles obtained from PAA directly correlates with concentrations of phosphate or sulfate anions and depends on the pH of the reaction solution [77,78]. Similar influences were also observed in the case of long chain polyamines (LCPAs) isolated from diatoms [54]. LCPAs isolated from diatoms are unique biomolecules and studying the structure-function relationship of synthetic mimics revealed an influence of alkyl chain length, number of amino groups and degree of methylation on silica precipitation activity and the morphology of silica material [79,166]. This understanding allowed the formation of hollow silica spheres and nonporous silica material [166]. Linear or branched polyethyleneimines (PEI) are simple polyamines but commonly used in biomimetic silica formation since they lead to almost exclusively spherical silica particles in phosphate containing buffer system [185]. The addition of methanol (70% v/v) leads to monodisperse silica spheres [186]. Different architectures of the PEI polymers or variation of the reaction conditions gave various silica materials, e.g., fibrils, flowers, plates, leafs and others [187,188,189,190]. Amine-terminated dendrimers were also used as variable templates for silica formation, in which the polypropylenimine-dendrimers (PPI) share the same momomeric units as the native LCPAs from diatoms [191]. Silica precipitating activity of amine-terminated dendrimers turned out to be dependent on the presence of phosphate anions and the size of the silica spheres can be controlled by phosphate concentration [192,193].

**Table 2:** Overview of silica structures obtained with different silica precipitating biomolecules.

	Silica Morphology		Conditions	References
**silaffin-1A**	spherical particles	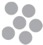	pH 5–5.5	[57]
**mixture of native silaffins**	cluster of small spheres	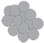	pH 5–5.5	[57]
**R5 peptide**	spherical particles	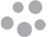	phosphate buffered solution, neutral pH, static conditions	[60,81,82,169,170]
	arch-shaped	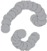	nitrogen stream bubbling through reaction mixture	[169]
	fibrillar	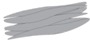	mechanical shear force; electrostatic/hydrodynamic force	[169]
	sheet-like	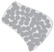	presence of polyhydroxy compounds (e.g., glycerol)	[170]
**poly-l-lysine (PLL)**	spherical particles	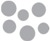	static conditions	[171,172]
	hexagons	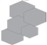	phosphate induced self-assembly of long-chain PLL	[173,174]
	fibrous	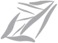	electrostatic field, long-chain PLL	[170]
	dendrite-like	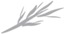	hydrodynamic field	[170]
**poly(l-cysteine_30_-b-l-lysine_200_)**	spheres	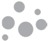	nitrogen atmosphere	[178]
	packed columns	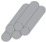	air-oxidation	
**lanreotide**	double-walled nanotubes	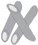	calcination of peptide template after silica formation	[180]
**A_6_K, V_6_K**	fibers	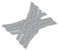	electrostatic field, flow field	[181]

#### 5.3.2. Biotechnological Applications Based on Biomimetic Silica

**Figure 8:**
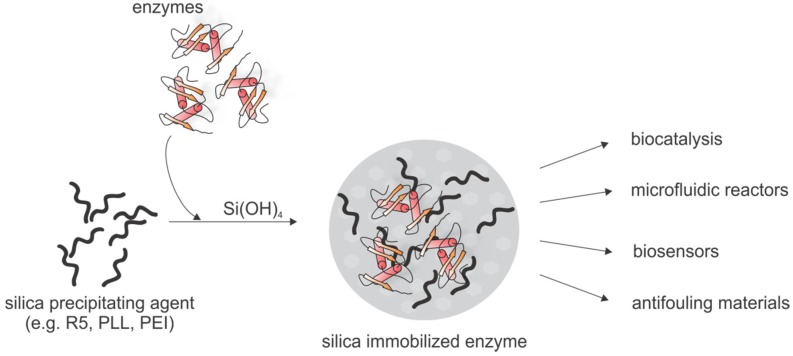
Schematic overview of enzyme immobilization via biomimetic silica formation and the application fields of silica immobilized enzymes.

The large variety of molecules with silica precipitating activity, the many options to influence the resulting silica structures and the mild reaction conditions led to development of diverse biotechnological applications based on biomimetic silica formation. A prominent application is immobilization of sensitive biomolecules such as enzymes (Figure 8). Generally, immobilization of biomolecules in mechanically stable and chemically inert silica matrices has the advantage of stabilizing the biomolecule, thereby often allowing transfer of these biomolecules to non-physiological environments and extend its lifetime, e.g., to make it reusable in a variety of applications such as biosensing, biocatalysis or drug delivery. Physical immobilization of biomolecules, *i.e.*, adsorption or entrapment of the biomolecule in a porous, insoluble matrix is often preferred over chemical immobilization via a covalent linkage, since it does not restrict the conformational freedom of biomolecules and therefore has less detrimental effects on protein function.

Enzyme immobilization in biomimetic silica has been achieved with different silica-precipitating agents, e.g., PEI [193,194,195,196,197], amine-terminated dendrimers [198], diethylenetriamine (DETA) [199], lysozyme [200,201,202,203], the cationic polysaccharide chitosan [204] or the R5 peptide [205,206]. Silica immobilization of the multimeric enzyme phenylalanine ammonia lyase was achieved with PEI, where coating of the enzyme with PEI prevented enzyme subunit dissociation and PEI served as silica precipitating agent [197].The enzymes typically become entrapped in the silica material with moderate to high efficiency while preserving enzymatic activity. However, not only the enzymatic activity of silica-entrapped enzymes can be rapidly assessed but also the structure using solid state NMR [207]. Confining enzymes in regular silica matrices could also be highly useful for structure elucidation using X-ray crystallography. Silica immobilized enzymes can serve as biocatalysts [205,206] and the simultaneous immobilization of multiple enzymes enabled the construction of a continuous silica biocatalyst device in which one enzyme recycles the cofactor for the other enzyme [196]. Another approach is based on immobilizing two coupled enzymes producing hydrogen peroxide. This silica-enzyme composite material was used to develop an enzyme based, environmental friendly anti-fouling paint for ship hulls [194]. For a similar application lysozyme, a cationic protein that was shown to be able to initiate silica formation [208,209], was incorporated into silica-lysozyme biocomposites that retain the antimicrobial properties of lysozyme and can be used as antifouling material as well [200].

The major drawback of immobilization approaches for enzymes in silica matrices based on co-precipitation during biomimetic silica formation is the random entrapment that may not ensure efficient and/or homogeneous encapsulation. These limitations can be overcome using covalent fusions of the R5 peptide to the target enzyme. This fusion can be achieved genetically and such protein-silaffin chimeras can initiate silica formation and result in controlled and efficient self-entrapment in the silica matrix [210,211,212]. The ability of self-entrapment of fusion proteins containing the R5 peptide has been exploited in the generation of biosensors [213,214]. Immobilization of carbonic anhydrase into bioinspired silica led to stabilization of this sensitive enzyme and opens the route for the development of an ecofriendly and efficient method to capture the greenhouse gas CO_2_ [199,212].

However, genetically fused and recombinantly expressed silaffin peptides lack the typical posttranslational modifications seen in diatoms and therefore major factors that control silica morphology and properties such as pore sizes are not accessible by this strategy. Recently, we could demonstrate how (posttranslational) modifications of synthetic R5 variants influence the formation of silica material with different morphologies [83]. Based on these findings a novel strategy for silica encapsulation of target proteins was established, in which chemically modified R5 peptides were covalently linked to target proteins through expressed protein ligation [215,216]. The resulting covalent conjugates of target proteins and modified R5 peptides precipitated silica efficiently and led to homogenous and functional encapsulation of the proteins in the resulting silica particles, highly superior to the random entrapment observed after simple co-precipitation [216].

In addition, covalent conjugation of other cargo such as small molecules and peptides to the synthetic R5 peptide allows efficient encapsulation of the cargo into biomimetic silica particles [83,217]. In combination with controlled release, e.g., by pH or redox sensitive linkers, useful delivery and/or reservoir materials based on biomimetic silica can be obtained. R5-cargo conjugates can be obtained via selective conjugation of thiol-functionalized cargo molecules, such as drug molecules, bioactive peptides or proteins, to a thiol-containing R5 peptide and result in high efficiencies of cargo encapsulation under mild conditions in a one-step procedure [217].

The advantageous mechanical properties of silica nanospheres also facilitate the application of entrapped enzymes in continuous flow-through reactors. Silica immobilized nitrobenzene nitroreductase was used to construct a microfluidic reactor for screening of cancer prodrug activation [195]. Another example showcases the immobilization of butyrylcholinesterase to screen the potency of cholinesterase inhibitors. Here a histidine-tagged R5 peptide variant was used to mediate selective binding to cobalt coated agarose beads. The R5-coated agarose mediated silica formation and enzyme encapsulation after addition of a silica precursor [218]. Such silica materials with different core structures should hold high potential for future use in biotechnology, bioimaging and medical applications [219].

The feasibility of silica deposition on planar surfaces has many potential practical uses. The R5 peptide has been used to deposit ordered arrays of silica nanospheres into a polymer hologram for construction of photonic devices [220]. Poly-l-lysine was also used for controlled patterned silica coating of surfaces under mild reaction conditions [221]. The integration of silica-encapsulated enzymes on planar surfaces empowers the generation of stabilized biosensors or enzyme microarrays and could be achieved with the R5 peptide or lysozyme [202,222]. The deposition of silica or a silica-enzyme layer on a gold surface mediated by lysoszyme increases the surface area and is therefore valuable for enhancing sensitivity in surface plasmon resonance spectroscopy applications [202]. Entrapment of enzymes in carbon-nanofiber silica composites provides a conductive matrix for the enzyme and gives rise to novel electrochemical biosensor systems [203,223,224].

## 6. Outlook

Overall, there is a multitude of very appealing applications of functionalized silica materials in biotechnology, medicine and in the controlled assembly of microscopic structure. Many recent examples are described above and especially the mild biomimetic silica variants will play a more important role in the near future. Applications of biomimetic silica that can be envisioned span the biotechnological use of tailor-made particles loaded with a specific (bio-) catalyst or combinations thereof that allow reusing of such valuable enzymes and installation of reaction chains for more complex transformations.

A renewed interest in core-shell particles that contain a core of gold (e.g., for imaging) or magnetite (e.g., for imaging or easy separation) and a porous shell of functionalized biomimetic silica would combine the best of two worlds in one particle. Medical application of biomimetic silica particles could also be further tweaked by using biocompatible but still fully controllable approaches to generate silica materials with controlled pore size and particle diameter. Such materials could be used as improved delivery tools for sensitive peptide and protein cargos in living systems.

Critical aspects that need to be considered are the overall size, homogeneity and stability of functionalized biomimetic silica materials that can vary depending on several parameters such as pH, ion strength, temperature, *etc.* during preparations and these need to be tightly controlled. Recent reports on potential toxic effects of silica nanoparticles [225,226] also need to be taken into account when developing new biomimetic silica materials for *in vivo* use. To this end, a recent debate about the contribution of peptide- and protein coronas assembling around nanoparticles, almost independent of the material used to create them, should be kept in mind since detrimental effects on particle function as well as an *in vivo* safety can be expected. Several publications have been addressing this point over the past few years [227,228,229].
